# Exploring care pathways of patients conveyed by emergency medical services (EMS) through electronic health records

**DOI:** 10.1186/s13049-025-01378-3

**Published:** 2025-04-09

**Authors:** Jani Paulin, Teijo I. Saari, Heikki Riihimäki, Mari Koivisto, Laura-Maria Peltonen

**Affiliations:** 1 Turku University of Applied Sciences and University of Turku, Turku, Finland; 2 Department of Anaesthesiology and Intensive Care, Division of Perioperative Services, Intensive Care and Pain Medicine, University of Turku, Turku University Hospital, Turku, Finland; 3 The wellbeing services county of Southwest Finland, Turku University of Applied Sciences, Turku, Finland; 4 Department of Biostatistics, University of Turku, Turku University Hospital, Turku, Finland; 5 Department of Health and Social Management, University of Eastern Finland and Wellbeing Services County of North Savo, Kuopio, Finland

**Keywords:** Prehospital emergency care, Emergency medical service, Conveyance, Subsequent event, Recontact, Care pathway

## Abstract

**Background:**

Emergency Medical Services (EMS) and Emergency Departments (ED) have reported increased patient volumes in the last decades. Despite high rates of non-conveyance decisions, unnecessary conveyances by EMS still occur. The aim of this study was to explore care pathways of conveyed patients by EMS through registry data.

**Methods:**

This was a retrospective cohort study of EMS patients in Finland. The primary outcomes were EMS recontacts and visits to a primary health care facility or ED within seven days. The secondary outcome was mortality within one week. Univariate and multivariable associations between the outcome variables and categorical variables were analysed with logistic regression. Results are presented with odds ratios (ORs) together with 95% confidence intervals (CIs) and *p*-values.

**Results:**

The conveyed patients’ visits to health care facilities were mainly brief. EMS arrival during night-time (20:00–08:00) (OR 1.69; 95% CI 1.59 to 1.80), in urban area (OR 1.21; 95% CI 1.13 to 1.29) and alcohol use (OR 2.55; 95% CI 2.26 to 2.86) predicted short ED visits (< 24 h). 77% of the patients were discharged from primary health care within one hour (median 22 min, IQR 18–60). After EMS conveyance and visit to the ED or primary health care facility, 10.5% of the patients were readmitted within one week. Non-urgent patients (OR 1.26; 95% CI 1.14 to 1.39), an EMS mission at night (OR 1.36; 95% CI 1.24 to 1.50), and based on univariate analyses, the usage of alcohol (OR 1.26; 95% CI 1.09 to 1.45) increased the likelihood of a readmission. 449 patients of all conveyed EMS patients (*n* = 20376) died within one week (2.2%).

**Conclusions:**

EMS non-conveyance reduces patient flow in EDs, but there is a possibility that more could be done related to unnecessary conveyances to health care facilities, especially in urban areas and at night. The pathway analyses of post conveyance re-contacts show that a small number of patients burden the system. Further in-depth studies are needed to understand of unnecessary conveyances, find solutions, and provide repeated users the appropriate care.

## Background

Patient volumes in prehospital emergency care [1, 2] and Emergency Departments (EDs) are increasing [3, 4]. Together with a global lack of healthcare resources [5], this poses challenge in delivering timely and sufficient services to all presented patients, and currently, an ambulance may be the only health care service available in many settings [6]. On one hand, the ageing populations, decreasing numbers of EDs and a centralization of health care services are associated with the increased patient volumes [1, 7]. On the other hand, dispatch over-triage [8] and a number of emergency medical services (EMS) patients without any need of medical intervention are known targets when seeking solutions to respond to crowding [2, 9]. Secondary telephone care assessment related to non-urgent patients was found to be a solution [10, 11] as well as community paramedic units [12, 13].

The role of prehospital emergency care has changed and increasing number of non-urgent patients are encountered without an immediate need for treatment in a health care facility [14]. Consequently, the Emergency Medical Services (EMS) have shifted towards providing on-site assessment and treatment, addressing the needs of the patients directly without compromising patient safety or conveyance into further care [15, 16]. Nevertheless, unnecessary conveyances by EMS are still reported [17, 18]. Reducing unnecessary conveyances is a key strategy to increase healthcare efficiency and optimal use of scarce resources.

Detecting deteriorating patients is a key part of prehospital emergency care [19]. Decision making related to care planning can be challenging due to the uncontrolled pre-hospital environment and unknown patient histories [20, 21]. EMS personnel’s’ decision-making requires flexible thinking, although some guidelines, protocols, warning scores, and supportive tools with new technology exist [22, 23]. Factors related to conveyance and non-conveyance decisions are indicated [e.g. 14, 18, 24, 25] and the decision to convey a patient or not is crucial for patient safety [26]. On the other hand, crowded EDs endanger patient safety as well [4, 27, 28]. Moreover, a lack of alternative care pathways has also been reported [29, 30].

Previously, the growing demand of EMS is partly explained by frequent users [31–33]. However, earlier research typically explored only one outcome or perspective related to frequent use of ED services. Little is known about the different types of recurrent contacts and care pathways seem to be unclear. The aim of this study was therefore to explore the care pathways of patients conveyed by EMS through the electronic health records data.

## Methods

### Setting

The main function of EMS is to provide care in urgent situations and if necessary, conveying the patient to the ED or primary care unit. In Finland, these services are provided by 21 wellbeing services counties as part of the healthcare system. The EMS completed 780 946 missions in Finland in 2023 (139 missions per 1000 inhabitants). The EMS are dispatched by the Emergency Response Centre (ERC), which operates the emergency number 112 in Finland. The ERC assigns each EMS mission a risk assessment class ranging from A to D, with A being the most urgent. A more urgent class takes priority over another in the mission response. The ERC is responsible for defining the appropriate EMS response to each emergency call, in accordance with the wellbeing services counties’ guidelines. The wellbeing services counties also define the level and content of the EMS in each county. This includes geographical risk assessment as well as EMS treatment and qualification guidelines. Finland has a multi-tier EMS unit system ranging from the first responder units to medical helicopters with a physician on board. The main EMS units are divided into basic life support (BLS) and advanced life support (ALS) ambulances. ALS units are the most common ones crewed with at least one advanced level paramedic-nurse with four years of education passing a bachelor’s degree (so called dual degree; paramedic and registered nurse qualification). These units can make non-conveyance decisions independently, where the National Health Care Act provides the legal basis.

### EMS data

**Fig. 1:**
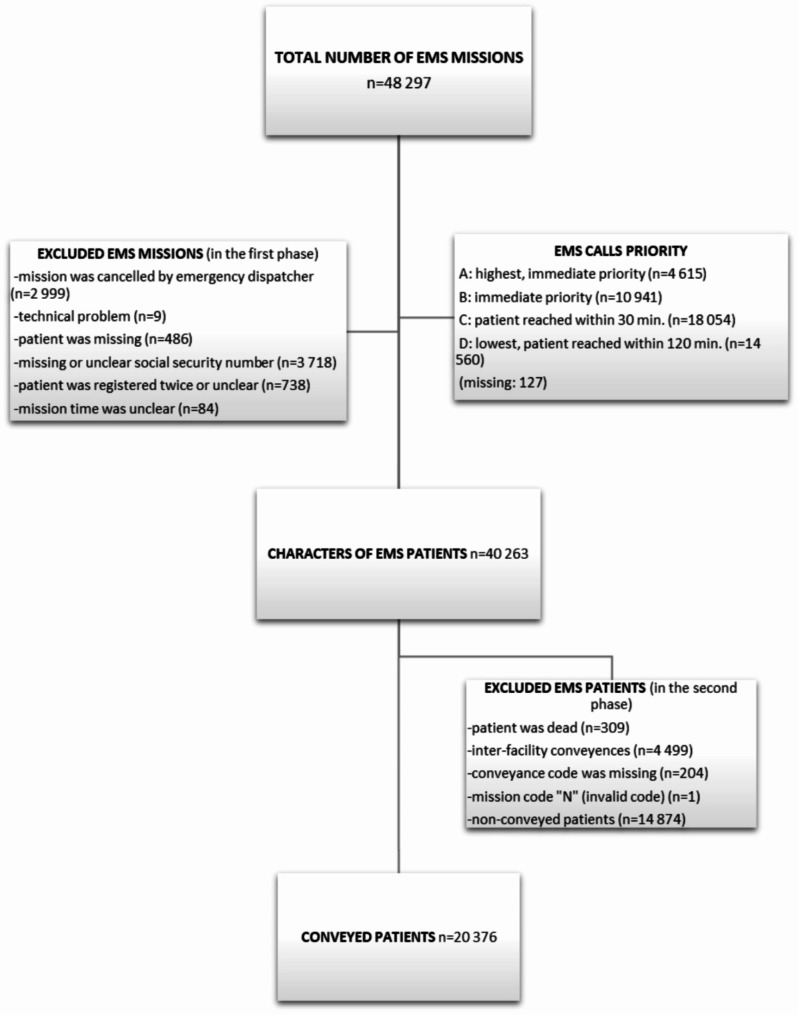
Flow chart

The EMS data was collected 1.6.2018–30.11.2018 from three wellbeing services counties (Kanta-Häme, Päijät-Häme, and South-Savo) in Finland (Fig. 1). The setting consisted of both urban and rural areas covering about 8.8% of the Finnish population with 482,805 inhabitants. The population density average is 26.1 people per square kilometer. The electronic patient care reporting (ePCRs) used by EMS personnel included many patients and missions related variables (e.g. personal identity number, age, gender, mission time and address, dispatch priority and code, vital signs like pulse rate, blood pressure, breathing rate and sounds, oxygen saturation, and narrative text sections). A detailed description of the ePCRs, urban-rural classification, the International Classification of Primary Care (ICPC2) as a main reason for care, NEWS2 scores, and alcohol usage were described previously [14].

### Data analysis

All patients who were conveyed to a health care facility after the EMS care needs assessment and treatment (*n* = 20,376) were included in the analyses (Fig. 1). Electronic health records data from various registries were merged with unique 10-digit personal identification numbers. The subsequent events were recorded for one week. Whether the patient’s EMS re-contact led to a conveyance, a new follow-up period was initiated. In Finland, the wellbeing services counties are legally required to submit care notifications of all treatment periods to registries of the Finnish Institute for Health and Welfare [34, 35]. Information of these registries included duration of visits and units, for example. It was not recorded whether the patient went to the health care facility by ambulance or by other means. In this study, all the EMS patients scheduled and unscheduled visits to primary health care or EDs were collected from all the wellbeing services counties. If the exact visit time was missing and the patient had a subsequent visit during the same day as the initial EMS conveyance mission, the EMS mission was set to be the first. The deceased patients were evaluated using the Finnish Causes of Death registry [36], which includes 100% of deceased patients in Finland during the calendar year. As above, a more comprehensive description of registries and data collection were described in previous work [16]. Moreover, related to conveyed patients, prior research reports that 1 in 3 (35.2%) missions were urgent with light and sirens, half of the missions occurred during daytime (47.1%), 68.2% occurred in urban areas, and 79.2% were operated by ALS units. The patients’ median age was 72 (IQR 53–83) and 51.5% were females. The decision to convey or discharge the patient at the scene was mostly made by the EMS units’ personnel. An EMS physician was rarely present at the missions (0.8%) and physicians were consulted in 18% of the cases [37].

### Outcome measures

The primary outcomes were EMS recontact and scheduled or unscheduled visits to a primary health care facility or ED for one week to find subsequent events after an EMS personnel’s conveyance decision. The secondary outcome was one week mortality.

### Statistical analyses

All statistical analyses were conducted using SAS for Windows, version 9.4. (SAS Institute Inc., Cary, NC, USA). Categorical variables were described using frequencies and percentages, and continuous variables as medians and interquartile ranges (IQRs). Univariate associations between the outcome variables and categorical study variables were analysed by using logistic regression. Multivariable logistic regression analysis included variables that were clinically and statistically significant in univariate analysis. Results are shown with odds ratios (ORs) together with 95% confidence intervals (CIs) and *p*-values, where *p*-value < 0.05 was considered statistically significant. The age groups were defined according to the Finnish national classification provided by Statistics Finland.

## Results

Of all the conveyed EMS patients (*n* = 20376) (Fig. 1), a total of 18,807 were treated in Eds and 12,144 in primary health care (including 10731 patients, which were treated both EDs and primary health care, missing *n* = 156). Overall, 449 patients died during the first seven days (2.2%). Altogether 1479 patients needed intensive care or treatment in high dependency unit. ICU-treatment was associated with increasing NEWS2 score calculated by EMS (OR 1.13; 95% CI 1.11 to 1.16) and EMS conveyance with urgency (OR 1.46; 95% CI 1.31 to 1.62).

The visits to the EDs were mainly short as 50% were discharged within 24 h. Based on the multivariable analyses, EMS arrival during night-time (20:00–08:00) (OR 1.69; 95% CI 1.59 to 1.80), EMS mission in an urban area (OR 1.21; 95% CI 1.13 to 1.29) and usage of alcohol by the patient (OR 2.55; 95% CI 2.26 to 2.86) predicted short visits (< 24 h) in the ED (Table 1). In contrast, based on univariate analyses, older age (OR 1.02; 95% CI 1.020 to 1.023) and each additional NEWS2 point increased the likelihood of a longer ED visits (> 24 h) (OR 1.11; 95% CI 1.10 to 1.13).

The visits to primary health care were partly brief and 77% of the patients were discharged within one hour (median 22 min, IQR 18–60). EMS mission in urban area (OR 2.27; 95% CI 1.89 to 2.74) and EMS arrival at night-time (20:00–08:00) (OR 1.54; 95% CI 1.22 to 1.93) increased the likelihood of short visit in primary health care units (Table 1).

After EMS conveyance and visit in the ED or primary health care facility, 10.5% of the patients had new health care contacts during the one week follow up period. The types of the recontacts varied between patients, and multiple types were seen. EMS reattendance and ED attendance were the most common types (Table 2). The patients with recontacts had a median age of 72 (IQR 54–84) and a median NEWS2 score of 1 (IQR 0–3) at the initial EMS mission. The most common initial ICPC2-codes chosen by EMS personnel were general weakness/tiredness, other psychological symptom/complaint, shortness of breath/dyspnoe, and acute abdomen (Table 3).

The recontacts were more likely to occur with non-urgent patients (OR 1.26; 95% CI 1.14 to 1.39), after EMS mission at nights (OR 1.36; 95% CI 1.24 to 1.50), and based on univariate analyses, when the patient had used alcohol (OR 1.26; 95% CI 1.09 to 1.45) (Table 1). Moreover, there was an association with patient’s older age and recontacts (OR 1.002; 95% CI 1.002 to 1.004). However, children’s subsequent events were rare (15–64 vs. < 15 OR 1.75; 95% CI 1.21 to 2.52, 65–84 vs. < 15 OR 1.77; 95% CI 1.23 to 2.55, > 85 vs. < 15 OR 1.88; 95% CI 1.30 to 2.72). The geographic location (*p* = 0.116) and the NEWS2 score (*p* = 0.946) did not predict recontact during the one week follow up period.

**Table 1:** Multivariable logistic regression analyses of short ED and primary health care visits and recontacts

Short ED visits							
	Missing	Univariate			Multivariate		
		OR (95%)	95% CI	p	OR	95% CI	p
Urban vs. rural	786	1.223	1.148–1.302	< 0.001	1.206	1.131–1.286	< 0.001
EMS arrival time							
20:00–8:00 vs. 8:00–20:00	28	1.814	1.706–1.929	< 0.001	1.692	1.588–1.803	< 0.001
Alcohol	28	2.871	2.562–3.217	< 0.001	2.546	2.264–2.863	< 0.001
Visit time: 49% = <24 h	0						
Short primary health care visits							
Urban vs. rural	332	2.308	1.916–2.781	< 0.001	2.274	1.887–2.741	< 0.001
EMS arrival time							
20:00–8:00 vs. 8:00–20:00	9224*	1.630	1.307–2.033	< 0.001	1.535	1.220–1.931	< 0.001
Visit time: 77% = <1 h	9224*						
Recontacts in one week							
EMS arrival time							
20:00–8:00 vs. 8:00–20:00	2	1.367	1.246-1.500	< 0.001	1.361	1.239–1.495	< 0.001
Alcohol	1	1.255	1.085–1.453	0.002	1.145	0.987–1.328	0.074
Mission priority							
CD va AB	0	1.247	1.132–1.373	< 0.001	1.256	1.140–1.385	< 0.001

*Due to unclear discharge times these patients were excluded from the analyses

**Table 2:**
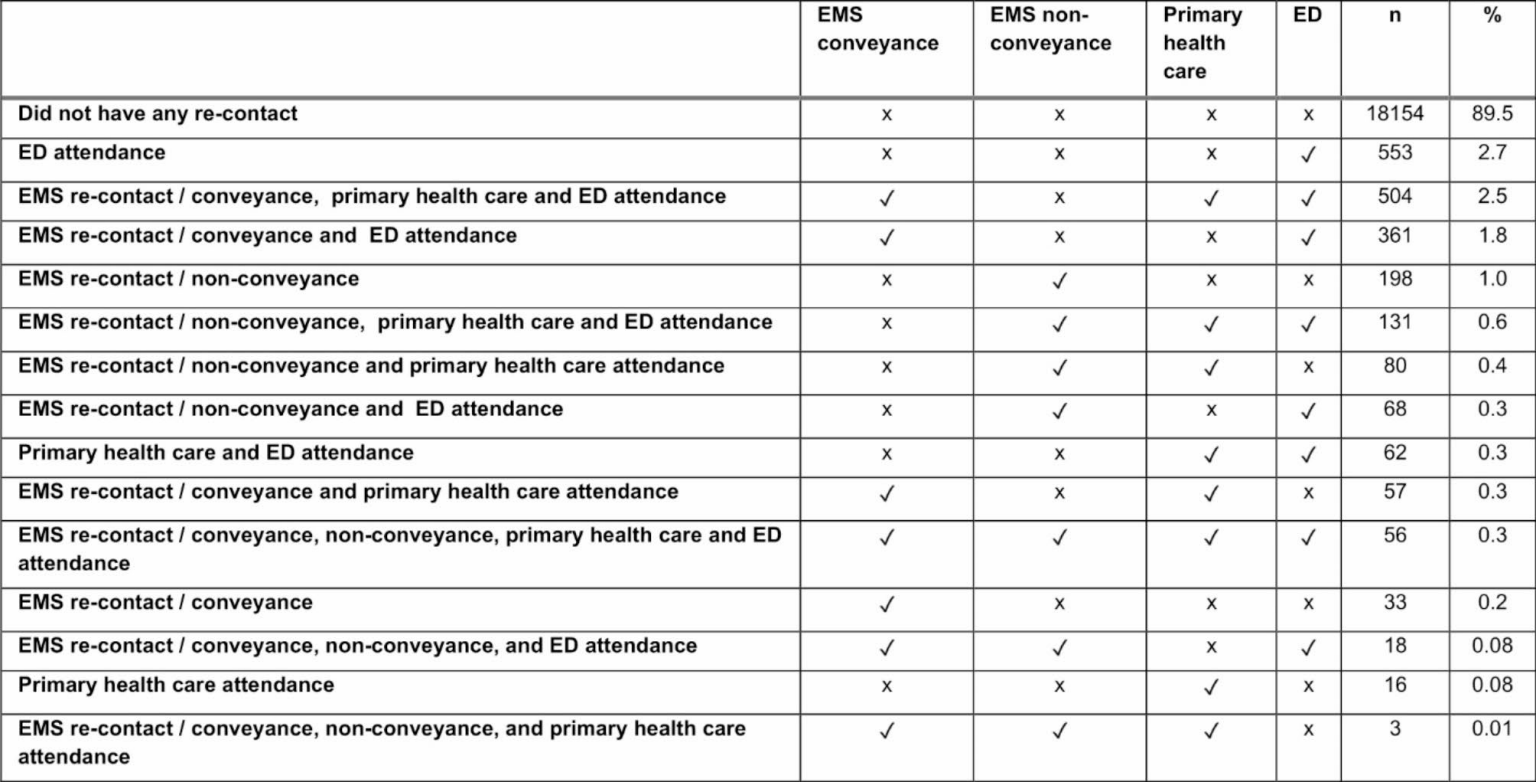
Pathway analyses of re-contacts in one week

**Table 3:** Conveyed patients’ initial, most frequent ICPC2–codes before re-contacts in one week

*n* = 19,208, missing 1086			
ICPC2		n	%
A04	Weakness/tiredness, general	2604	13.6
P29	Psychological symptom/complaint other	1039	5.4
R02	Shortness of breath/dyspnea	902	5.1
D01	Acute abdomen	855	4.5
A03	Fever	825	4.3
N80	Head trauma	660	3.4
K90	Stroke	641	3.3
K74	Iscaemic chest pain	594	3.1
N07	Convulsions/seizure	587	3.1
P16	Acute alcohol abuse	584	3.0

## Discussion

The main findings of our study are that the visits of the patients conveyed by the EMS to health care facilities were mainly brief, especially in the urban areas and during the night. Furthermore, one week follow-up period demonstrated that 10% of the patients recontacted health care providers with multiple types of contacts.

Traditionally, EMS treat high-risk patients with critical emergencies. Our study indicates that 7.3% of the conveyed patients needed intensive care or treatment in a high dependency unit. Understandably, NEWS2-scores and EMS conveyance with urgency predicted these cases. Overall, 1 out of 50 patients deceased during the one week follow up period, which is in line with previous studies [38, 39].

Our study demonstrates that the visits to the health care facilities after an EMS conveyance were mostly short, in 77% of the visits to primary care, the duration of the visit was less than an hour. Clearly, some of these patients were quickly transferred to specialized healthcare through an ED, and some brief visits may remain essential for patient care, such as those for minor procedures or wound management, but this raises the question, however, whether the conveyance decision was appropriate in all of these cases. According to the univariate analyses, the urban area and EMS arrival at night predicted short visits in the health care facilities. Although, patients’ perception might differ from the EMS perception [40], and lack of alternative care options are reported [30], and ED related factors clearly exist [41], there is a possibility that the EMS personnel sometimes decided to convey the patient with a lower threshold. Non-conveyance is more time consuming [14, 42], requires more a physician’s consultation [14, 43] and plans for further treatment in agreement with the patient [44]. Therefore, conveyance may be an easier option. Previous findings indicate that majority of conveyed patients are non-urgent, discharged directly without any need of hospital resources, and therefore are considered potentially avoidable [18]. Similarly, a Finnish study found that 3 out of 4 conveyed patients did not receive any medical treatment or diagnostic tests before the next morning [45]. We also found that alcohol usage was associated with short ED visits, which does not, however, automatically mean that the visit is unnecessary. For example, a wound of a drunken patient may be quickly treated. However, the balance between safety margins and limited resources is necessary and more studies are needed to explore the appropriate allocation of scarce resources [17].

Of all the patients conveyed by the EMS, most did not have any re-contacts during the follow-up period. However, our pathway analyses indicates that 1 out of 10 patients had a subsequent contact, some of which had several different ones. Similar results have been reported before but mainly based on a limited number of outcomes [31–33]. Our study reveals that EMS re-contact and ED attendance were the most common types of re-contacts. Obviously, some of these patients needed urgent treatment. One explanation may be limited access to primary care [7]. However, as mentioned earlier, reasons for a crowded ED are many, and it is potentially high-risk place for patients [41, 46]. In this study, the high number of non-conveyance EMS decisions after an EMS re-call indicate that some of the patient’s complaints were minor, or that there was not a need for treatment. That said, patients’ guidance on the role of primary care is seen important and requires more attention [47].

Typical reason for care chosen by the EMS personnel, was general weakness/tiredness, which has been previously found to be associated with adverse patient outcomes [39, 48]. The absence of an appropriate code in the documentation system might be a reason for the high use of the “general weakness” -code [14]. However, shortness of breath and acute abdomen were also common reasons for care and are more specific clinical problems and related to repeat user. Psychological symptom/complaint was in our material a common cause for EMS mission, known to be associated with frequent users [2, 31, 40]. Our study also highlights that non-urgent EMS missions, night-time admissions, older age, and alcohol use increased the likelihood of re-contacts, in accordance with prior literature [e.g. 2, 33, 49].

EMS patients do not necessary end up at the most suitable level of care [50]. For example, Strum et al. (2021) found that in case of EMS conveyance, the probability of hospital admission was fourfold [51]. It appears that patients conveyed by the EMS do not necessary receive the help they need leading to inappropriate use of different services. Hence, paying more attention to the care pathways becomes important to reduce unnecessary healthcare facility visits and better meet the care needs of individual patients.

### Limitations

Our study has limitations, such as missing data, excluded patients, ICPC2 classification and NEWS2 score were described before [14]. The data was collected few years ago, and after that clinical practices may have changed. For example, the EMS dispatch criteria have been updated to reduce unnecessary missions of EMS units. On the other hand, it is assumed that many of decreased missions would have ended up to EMS non-conveyance decision. However, the few years old data decreases the reliability of this study. This study has some clear strengths like sufficient sample size and possibility to follow patients between the registries although the ePCRs and registries that were used in this study were not initially created for scientific research purposes. For instance, the ED visit register included the date, but the specific time of the visit was mostly missing. There were issues related to discharge times in the data extracted from primary health care, which is why we had to exclude many patients from analyses related to visit times. Thus, there were challenges to analyse the exact visit times and patient flow. Furthermore, in some areas the EDs and primary health care units operate in the same premises, which might explain the large number of patients who had both ED visits and primary care visits. Clearly, some of these patients were quickly transferred to ED. A potential bias is related to missing exact visits times in the EDs. Whether the time was missing, the initial EMS mission was judged as the first, which may have increased the number of re-contacts. Our data does not include private clinics visits, which may affect the opposite way.

## Conclusions

Although appropriate EMS non-conveyance reduces the patient flow pressure in EDs, there is a possibility that more could be done to identify unnecessary conveyances into health care facilities, especially in urban area at night. However, the definition of unnecessary conveyances and their alternative explanations need to be clarified. The pathway analyses of post conveyance re-contacts indicates that there is a small proportion of patients that burden the system. More studies with in-depth analyses are needed to understand the reasons of unnecessary conveyances, find solutions, and provide repeated users the help they need.

## Data Availability

No datasets were generated or analysed during the current study.

## Abbreviations

EMS: Emergency medical services
ED: Emergency Department
NEWS2: National Early Warning Score
ALS: Advanced life support
ePCR: Electronic patient care reporting
ICPC2: International Classification of Primary Care, Second Edition
